# Thermal Variability Modulates Altitudinal Differences in Metabolic Plasticity of the Asiatic Toad

**DOI:** 10.1002/ece3.72319

**Published:** 2025-10-08

**Authors:** Yuechan Zhang, Song Tan, Jinzhong Fu, Jingfeng Chen

**Affiliations:** ^1^ CAS Key Laboratory of Mountain Ecological Restoration and Bioresource Utilization & Ecological Restoration and Biodiversity Conservation Key Laboratory of Sichuan Province, Chengdu Institute of Biology Chinese Academy of Sciences Chengdu China; ^2^ Department of Integrative Biology University of Guelph Guelph Ontario Canada

**Keywords:** altitudinal gradient, *Bufo gargarizans*, maximum metabolic rate, metabolic plasticity, resting metabolic rate

## Abstract

Physiological plasticity is crucial for survival in fluctuating environments. The climate variability hypothesis (CVH) proposes that physiological plasticity scales with climatic variation across geographical gradients, yet its generality remains debated. Furthermore, the mediating role of prior thermal history is largely unexplored. This study examines how altitude and thermal variation shape phenotypic plasticity in resting metabolic rate (RMR) and maximum metabolic rate (MMR) of Asiatic toads ( *Bufo gargarizans* ). We found that RMR plasticity, but not MMR plasticity, varied altitudinally and was influenced by thermal conditions. Compared to low‐altitude toads, high‐altitude individuals exhibited reduced RMR plasticity, contradicting the CVH. This difference was amplified under higher thermal variability. In contrast, MMR plasticity showed no altitudinal variation or response to thermal variability. However, warm acclimation significantly increased MMR thermal sensitivity. Metabolic substrate choice depended on pre‐acclimation thermal experience. Our results indicate that RMR plasticity, rather than MMR plasticity, primarily underpins altitudinal adaptation, and increased thermal fluctuation may disrupt this adaptive pattern. This research provides novel insights into macrophysiological responses to global warming.

## Graphical abstract

This study shows that the plasticity of the metabolic floor, but not the metabolic ceiling, varies along altitudinal gradients and is influenced by the thermal context prior to thermal acclimatization in *Bufo gargarizans* . The data suggest that maintenance of metabolic plasticity is likely to be the primary strategy for adaptation to altitudinal gradients and that increased thermal variability due to climate warming may disrupt this adaptive pattern.

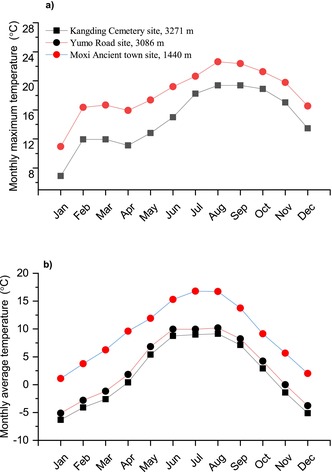

## 1 Introduction

The swift rate of alteration in Earth's climate is unprecedented and carries significant consequences for biodiversity. In the context of climate change, the survival of a species or population hinges on its ability to alleviate thermal stress, whether via migration, genetic adaptation, or acclimation (Huey et al. 2012). Given the limited dispersal capacity and the gradual development of genetic adaptation, the physiological plasticity of ectotherms is arguably the paramount mechanism for addressing global warming (Hoffmann and Sgro 2011; Seebacher et al. 2015; Rohr et al. 2018; Barley et al. 2021). Given that selection driven by historical thermal regimes shapes physiological plasticity, it is imperative to elucidate the variations in physiological plasticity across diverse geographical regions (Huey et al. 2012; Gunderson and Stillman 2015; Norin and Metcalfe 2019).

The metabolic rate, akin to several physiological characteristics, demonstrates phenotypic plasticity in reaction to alterations in the animal's internal condition or its surroundings (Norin and Metcalfe 2019; Sun et al. 2022). Metabolic alterations can be orchestrated to enable ectotherms to manage anticipated fluctuations in their energy state or requirements. The climate variability hypothesis (CVH) posits that climatic variations along altitudinal and latitudinal gradients should result in comparable adaptations in physiological tolerance and adaptability (Ghalambor et al. 2006; Sheldon et al. 2018). However, an analytical review has shown that, in contrast to freshwater animals, terrestrial animals from thermally stable environments (tropical zone) have a greater metabolic capacity for metabolic acclimation than animals from variable environments (temperate zone) (Seebacher et al. 2015). It remains unclear whether this observation is generally applicable to other spatial thermal gradients (e.g., altitudinal, urbanization), which differ in several aspects of thermal characteristics (e.g., thermal slopes) and selective forces (Todgham and Stillman 2013; Verheyen et al. 2019; Christian et al. 2024).

Under present global warming scenarios, temporal thermal variability also impacts metabolic plasticity and individual fitness in addition to thermal variability at the regional scales (Bozinovic and Portner 2015). The beneficial acclimation hypothesis (BAH) posits that organismal performance may decline during a thermally variable phase subsequent to a stable temperature period. This is because they need time to adjust to the altered thermal environment (Huey et al. 1999; Bernhardt et al. 2020). However, organisms capable of quick acclimation in response to environmental changes may lessen such adverse times. Conversely, under a “jack of all trades, master of some” framework (sensu Richards et al. 2006), historical thermal variation may yield advantageous effects on performance during subsequent prolonged temperature exposure, as prior acclimation to thermal fluctuations can be generally beneficial in novel, sublethal thermal surroundings. A recent meta‐analysis shows that the effects of thermal variability, either under acclimation or acute experimental designs, are highly dependent on the properties of organisms and the thermal regime they have experienced (Slein et al. 2023). Nevertheless, most of the studies analyzed have focused on the metabolic performances of organisms themselves (e.g., growth rate, sprint speed, cytochrome c oxidase activity) but not on the plasticity of these performance traits. Therefore, it remains unknown whether increasing thermal variability prior to acclimation is beneficial or detrimental to the acclimation capacity of energy metabolism. Furthermore, we have not tested whether temporal and spatial thermal variability influence metabolic plasticity together or independently.

Resting metabolic rate (RMR) and maximum metabolic rate (MMR) are the minimum and maximum energy requirements for basic homeostasis functions and multiple physiological processes like locomotion, digestion, reproduction, and thermoregulation, respectively (Auer et al. 2017). RMR represents the cost of maintaining the metabolic apparatus needed to support MMR; hence, these two metabolic variables are generally positively connected. A recent study found that they are not connected across individuals and are not associated with the same mitochondrial functional characteristic (Salin et al. 2016). They also respond differently to thermal variability: as temperature rises, their metabolic floor (RMR) becomes more malleable than their ceiling (MMR) (Sandblom et al. 2016), while the reverse pattern has also been seen (Rodgers and Franklin 2021). These findings suggest that the metabolic floor and ceiling are controlled by distinct physiological processes, and defining the plasticity of these two metabolic features would provide a better understanding of their adaptive significance in coping with spatiotemporal thermal variability.

The Asiatic toad ( *Bufo gargarizans* ) is an ecological generalist extensively found over East Asia, ranging from sea level to the Qinhai–Tibetan Plateau (Fei et al. 2009). Several functional categories related to energy metabolism were found to have a high concentration of genes with plastic expression differences in juvenile toads, according to a previous reciprocal transplantation experiment (Yang et al. 2017). This suggests that metabolic plasticity could be important for this species' thermal adaptation to high altitude. A recent study also showed that the acclimation capacity of RMR in adult toads is lower at high altitudes than at low altitudes (790–2010 m), demonstrating that greater temperature variability at high altitude does indeed favor a less plastic phenotype at the whole organism level Tan et al. (2024). However, this work did not distinguish between active and passive plasticity, which can be assessed only using a fully factorial “acclimation and acute” experimental design (Havird et al. 2020). Therefore, in this study, we aim to verify the effects of altitude on the active plasticity of RMR and MMR of *B. gargarizans* and to investigate how pre‐acclimation thermal variability would modify these effects. We predict that toads at high altitude will have low plasticity of RMR but not MMR. We also predict that pre‐acclimation thermal variability will exacerbate the detrimental effects of altitude on metabolic plasticity.

## 2 Materials and Methods

### 2.1 Collections of Animals

**FIGURE 1:**
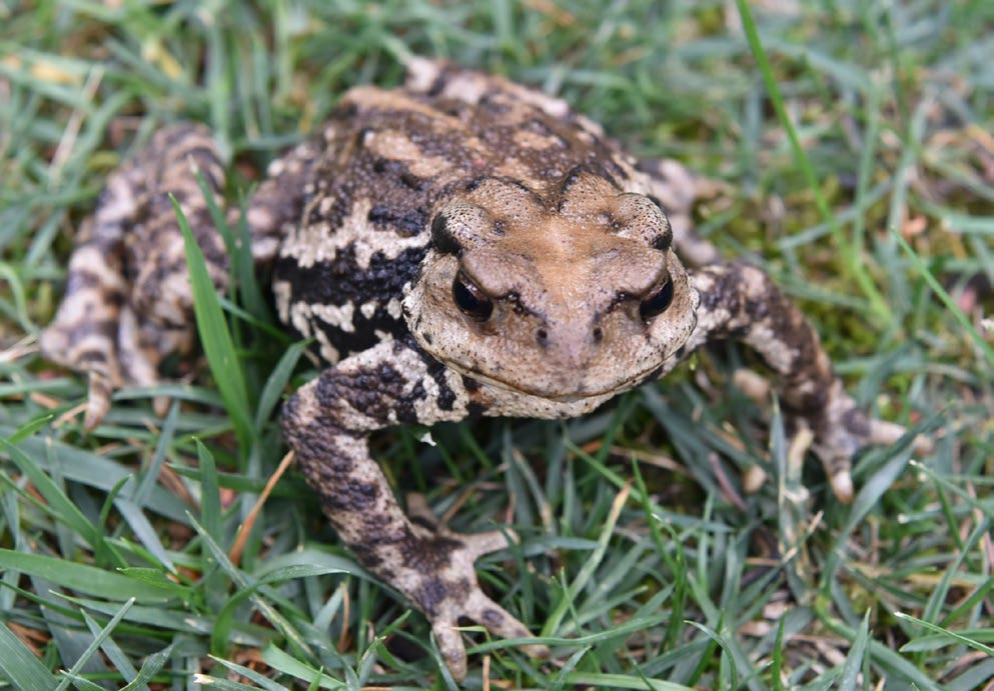
A photograph of a male Asiatic toad ( *Bufo gargarizans* ) captured in Moxi Ancient Town.

**FIGURE 2:**
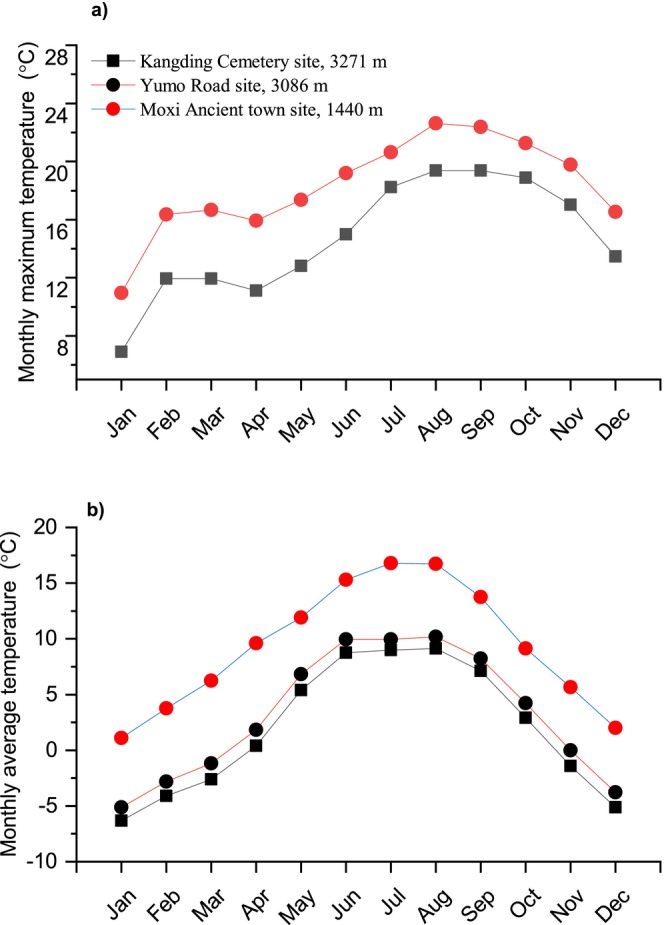
Monthly maximum temperatures and monthly average air temperatures from 2012 to 2019 at the three sampling locations. Low‐altitude location: Moxi Ancient Town; high‐altitude locations: Kangding Cemetery site and Yumo Road site.

Sixty adult male *B. gargarizans* (Figure 1) were collected from three locations in the western Chinese Hengduan Mountains during the breeding season of 2022 (Table S1). One site is located at an elevation of 1440 m (Moxi Ancient town) and is considered a low elevation population (LA population); the other two were located at 3086 m (Yumo Road) and at 3271 m (Kangding Cemetery) and are considered a high elevation population (HA population). Thirty animals were sampled from either the high or low altitudes. The straight distance between the LA population and HA population does not exceed 50 km. The two locations share a river valley. Figure 2 shows that throughout the year the LA population has an average monthly temperature that is 5°C higher than the HA population. Forests with evergreen foliage are typical of the flora in both locations. We did not measure the toads' food supplies (arthropod) at either location, but according to Liang et al. (2018), their diversity and abundance usually fluctuate along an elevation gradient. Our lab at the Chengdu Institute of Biology, Chinese Academy of Sciences (CAS) (an elevation of 457 m) received all of the toads. Each toad was kept in its own plastic container in the laboratory, measuring 35.5 × 25 × 15 cm (length, width, and height). They were subjected to a consistent photoperiod of 12 h of light and 12 h of dark cycles. Toads were provided with crickets and mealworms dusted in calcium powder (Exo Terra) ad libitum during the acclimation and re‐acclimation phases. Every 2 days, their meal dishes were emptied and replaced with fresh ones. To conserve water and provide refuge, each container also contained a piece of wet sponge measuring 5 × 7 cm and a U‐shaped tile measuring 5 × 7 cm. To minimize the effects of thermal stress, all the toads were initially placed at 10°C, and then the ambient temperature was raised to 21°C with an increase of 1°C every 2 days. The acclimation temperature corresponds to the monthly maximum temperature between June and July (Figure 2). All animal procedures were carried out in accordance with approved protocols from the Animal Care and Use Committee at the Chengdu Institute of Biology, CAS.

### 2.2 Acclimation Schedule

**FIGURE 3:**
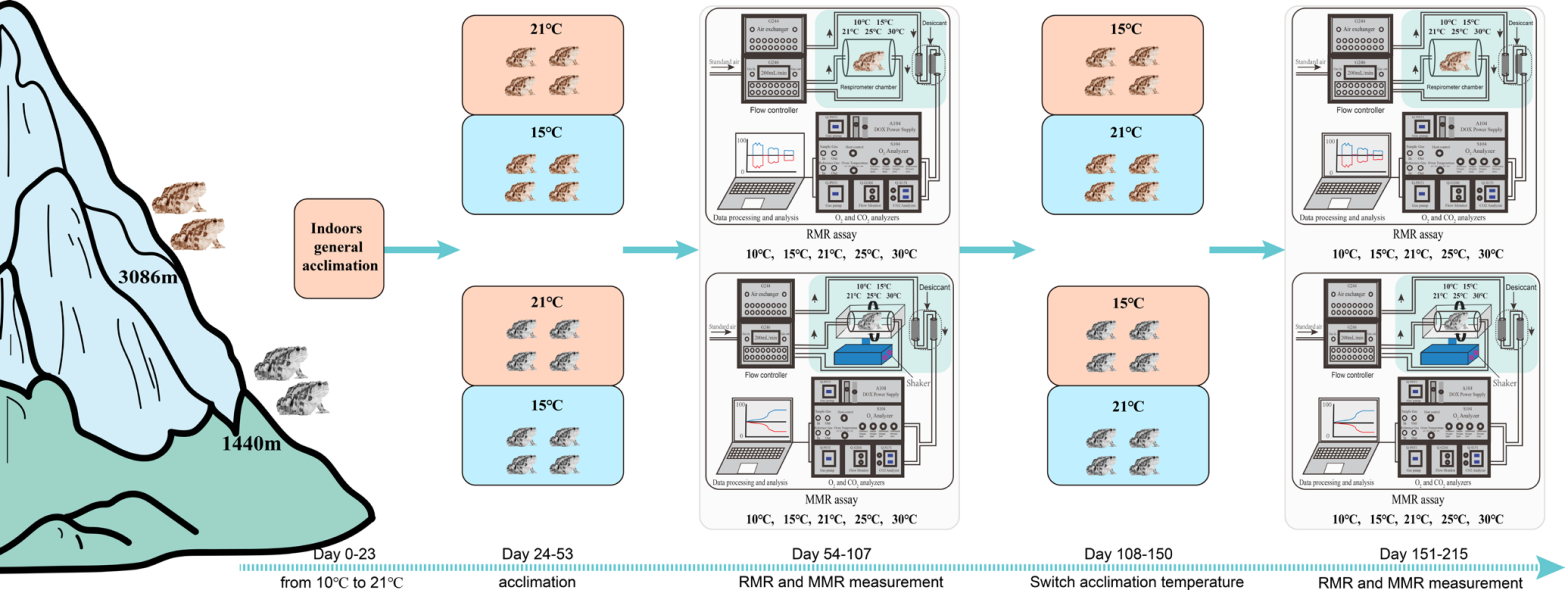
Timeline of the experiment. Days 0–23, captured toads were placed at 10°C, and then the ambient temperature was raised to 21°C, increasing by 1°C every 2 days. Days 24–53, the toads from each altitude were acclimated at 21°C or 15°C. Days 54–107, resting/maximum metabolic rates were measured for all toads at five test temperatures (10°C, 15°C, 21°C, 25°C, 30°C). Days 108–150, all toads were acclimated to switched ambient temperature. Days 151–215, all toads were measured again for resting/maximum metabolic rates at five test temperatures (10°C, 15°C, 21°C, 25°C, 30°C).

After 23 days of acclimation (Days 0–22, Figure 3), all toads from each altitudinal population were ranked by their body mass and alternatively assigned to two thermal settings (21°C and 15°C) and acclimated for 30 days (Days 23–52). In both low‐ and high‐population scenarios, we designate 15°C and 21°C as the thermal regimes for spring (March–May) and summer (June–August) in common garden environments. The thermal circumstances do not precisely represent the absolute maximum temperature of either population, which facilitates the identification of the genetic underpinning of metabolic adaptability. All subjects were then measured for RMR and MMR (last 55 days, Days 53–117). After the first round of metabolic measurements, the toads acclimated to 21°C were subjected to a 30‐day acclimation to 15°C (Days 139–193), and similarly, the toads acclimated to 15°C were subjected to a 30‐day acclimation to 21°C. During the temperature transition period (Days 117–139), we followed the same graduation rules: increase or decrease by 1°C every 2 days. At the end of the switched acclimation, the RMR and MMR of all toads were measured again. With this acclimation setting, the group of toads initially acclimated at 15°C experienced greater thermal variability than those initially acclimated at 21°C. Furthermore, this within‐individual design avoids the problem of genetic heterogeneity in the replicated‐individual design and facilitates the interpretation of metabolic plasticity (Stamps 2016). All toads were healthy throughout the experiment. They were returned to their capture sites and released.

### 2.3 Oxygen Consumption Measurement

Metabolic rates were assessed utilizing an automated flow‐through respirometry device as previously conducted (TSE device, Germany) Tan et al. (2022; Tan et al. 2021). The mating seasons of the LA and HA populations are separated by a 3‐month interval, resulting in nonoverlapping measurement periods. For each population, the resting metabolic rate (RMR) and maximum metabolic rate (MMR) of 30 toads were assessed throughout five trials, corresponding to five randomized temperature settings (10°C, 15°C, 20°C, 25°C, and 30°C). In each experiment, the 30 toads were allocated into eight groups (one group each day), comprising four toads per group for the initial seven groups and two toads per group for the last group, given that four sampling chambers were accessible in the respirometry apparatus. We also randomized the sequencing of the batches, maintaining the same order for each trial. To mitigate the systematic bias resulting from the extended duration of the metabolic assessment, we organized each batch to consist of an equal number of subjects from the 15°C acclimation group and the 21°C acclimation group.

RMR was initially measured in each batch, subsequently followed by MMR. The toads underwent a fasting period of 5 days prior to each RMR/MMR measurement and were allowed to rest for 5 days following each measurement. Metabolic measures were conducted from 7 am to 11 pm. We precisely measured the body mass of each toad and thereafter placed them into a cylindrical metabolic chamber (900 mL), where the ambient temperature was regulated at the test temperature ±1°C (mean ± SE) using a biochemical incubator (model: LRH‐250F, Yi‐Heng, China). A little, damp face tissue was placed within the metabolic chamber to avert desiccation. The source gas was sent through a magnesium perchlorate column before entering a mass flow controller that regulated the flow into the metabolic chambers. The flow rate was established at 200 mL/min. The airflow leaving the chambers entered a gas switcher, which channeled the air from a focal chamber via the gas analyzers. The effluent gas stream was concurrently subsampled through H_2_O scrubbers before entering an O_2_ and CO_2_ analyzer (Tan et al. 2022). Oxygen consumption was routinely documented for 5 h throughout each attempt at resting metabolic rate monitoring. Animals were measured in succession, with the reference chamber measured between each animal. Each cycle lasted 36 min, comprising 12 min of recording two animals and 6 min of recording the reference chamber. We acquired eight measures for each animal over the 5‐h duration. The initial 60 s of each measurement were omitted to enable the system to equilibrate after transitioning between chambers, and we computed RMR as the average of 180 s (from the remaining 300 s of the measurement period) of the lowest continuous stable oxygen recordings across the eight cycles.

In accordance with the literature, we created a motorized rotating device to measure toad MMR (Taigen and Pough 1983; Careau et al. 2014). The metabolic chamber was set up on a specially designed digital rocking shaker (model: SK‐R330‐Pro, Da‐Long, China) that rotated at about two revolutions per minute. MMR was recorded until the observed oxygen‐consumption curve plateaued while the toads were repeatedly driven to correct their body positions. MMR was proxied by the highest consistent 3‐min interval of oxygen consumption. The body mass of each toad was assessed both before and after metabolic measurements, and the allometric correction of oxygen consumption was calculated using the average. The difference between MMR and RMR was used to compute net aerobic scope (NAS), and the ratio of CO_2_ production to oxygen consumption was used to determine the respiratory quotient (RQ).

### 2.4 Data Analysis

The effects of elevation (Elev), acclimation temperature (Ta), sequence of acclimation temperature (SE), test temperature (Tt), sequence of test temperature (Se), experimental trial, and body mass on RMR, MMR, NAS, and RQ were detected using a univariate linear model. The interindividual covariations between RMR and MMR were examined using a bivariate mixed model. The R package “MCMCglmm” was utilized (Hadfield 2010). To help the models converge, all continuous variables were scaled (mean = 0, SD = 1) and standardized using the Johnson transformation (Minitab 16.0) (Schielzeth 2010).

Subject identification was set as a random effect, body mass was entered as a covariate (not included when body mass itself was used as a response variable), and Elev, Ta, Se, Tt, St, Round, and/or their interaction were entered as categorical fixed factors for each univariate linear model. No variation in size scaling between populations was observed in our earlier investigation (Tan et al. 2021). As a result, we did not fit a distinct body mass slope for every population. Several candidate models were examined and simplified using the deviance information criterion (DIC) and stepwise backwards elimination for each response variable. The priors for random effects and residuals were set to *V* = 1 and nu = 0.002, respectively, when metabolic variables were examined. Body mass was entered as a covariate and subject identity as a random effect for every bivariate mixed model. We did not take into account how those categorical fixed factors affected the relationships between these variables because of the limited sample size. Assuming (conditional) inverse‐Wishart prior distributions, the priors for random effects and residuals were independently set as *V* = diag (2), nu = 1.002 and *V* = diag (2), fixed = 1 (Hadfield 2010). The model was run for 300,000 iterations with a burn‐in of 30,000 and a thinning interval of 100 in order to calculate the posterior distribution. This resulted in an effective sample size of 2700 with an autocorrelation level below 0.05. The posterior distributions provided us with trait repeatability (*R*) and its 95% CI (*R* = between‐individual variance/[between‐individual variance + within‐individual variance]; Dingemanse and Dochtermann 2013).

## 3 Results

### 3.1 Body Mass

Body mass increased continuously throughout the experiment (*p*_round_ < 0.001, Tables S2 and S3), suggesting that the toads' body condition was well maintained. For the toads from low altitudes, body mass acclimated at 21°C was significantly higher than at 15°C (Figure S1, Table S3). Moreover, the difference increased as the thermal variability of acclimation increased (i.e., in the acclimation sequence, first acclimated at 15°C then at 21°C). In contrast, acclimation temperature and acclimation thermal variability had no effect on the body mass of toads from high altitudes. In addition, there was a slight increase in body mass as the test temperature increased, which could be a carry‐over effect of metabolic measurements on food intake.

### 3.2 RMR, MMR, and Their Associations

**FIGURE 4:**
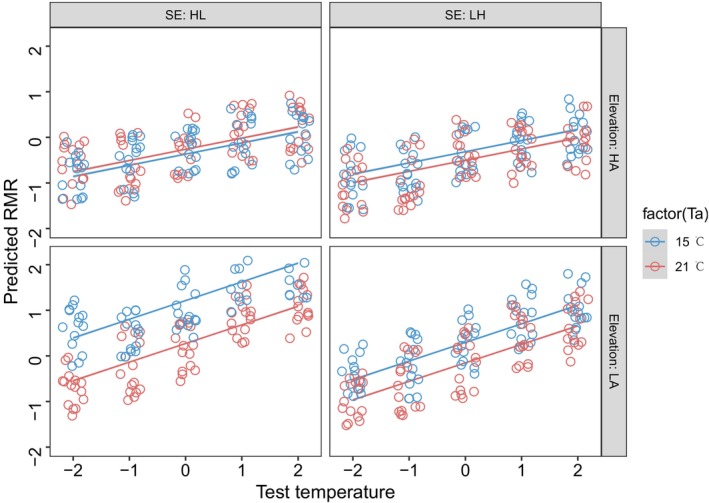
The effects of acclimation temperature, acclimation sequence, test temperature, and altitude on the resting metabolic rate (RMR) of the *Bufo gargarizans* . Top left: The high‐altitude group acclimated first at 21°C and then at 15°C. Top right: The high‐altitude group acclimated first at 15°C and then at 21°C. Bottom left: The low‐altitude group acclimated first at 21°C and then at 15°C. Bottom right: The high‐altitude group acclimated first at 15°C and then at 21°C. The blue or red dots represent the predicted value of RMR at 15°C and 21°C acclimations respectively. The blue or red lines are the regression lines based on the predicted RMR.

RMR increased with increasing test temperature in both altitude populations, and the rate of increase was higher in the low‐altitude population (Figure 4, Tables S4 and S5). Only the toads from low altitudes were sensitive to the cold acclimation (15°C). Moreover, for the toads from low altitudes, the increase in RMR was more significant in those toads acclimated first at 21°C and then at 15°C. Furthermore, body mass was not associated with RMR. The conditional repeatability of RMR was moderate (0.274 [0.163, 0.362]).

**FIGURE 5:**
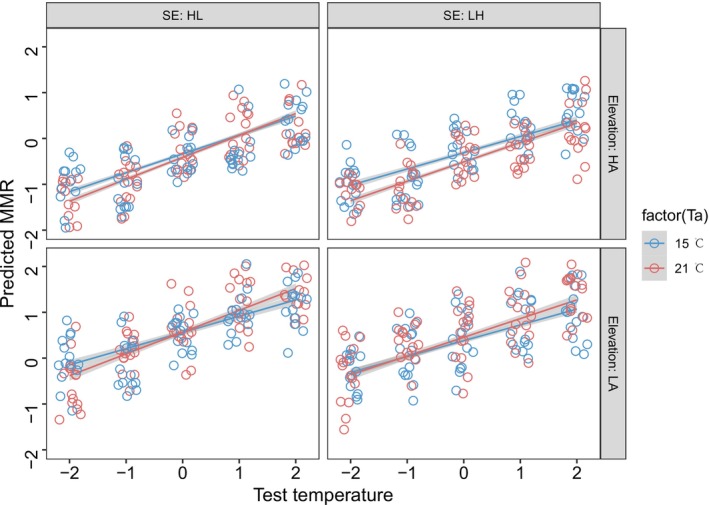
The effects of acclimation temperature, acclimation sequence, test temperature, and altitude on maximum metabolic rate (MMR) of *Bufo gargarizans* . Top left: The high‐altitude group acclimated first at 21°C and then at 15°C. Top right: The high‐altitude group acclimated first at 15°C and then at 21°C. Bottom left: The low‐altitude group acclimated first at 21°C and then at 15°C. Bottom right: The high‐altitude group acclimated first at 15°C and then at 21°C. The blue or red dots represent the predicted value of RMR at 15°C and 21°C acclimations respectively. The blue or red lines are the regression lines based on the predicted MMR.

The MMR of low‐altitude toads was generally higher than that of the high‐altitude toads (Figure 5, Tables S6 and S7). Under 21°C acclimation, the MMR was more sensitive to increased test temperature than under 15°C acclimation. In each measurement round, the sequence of test temperatures had a negative effect on MMR. MMR was significantly correlated with body mass. The conditional repeatability of MMR was moderate (0.396 [0.280, 0.507]). There was no correlation between MMR and RMR at the interindividual level (0.028 [−0.029, 0.093]), while the correlation between them at the intraindividual level was significant (0.360 [0.294, 0.429]) (Table S8).

### 3.3 Aerobic Scope, RQ

For the toads acclimated first at 21°C and then at 15°C, when the thermal dependence of NAS was varied with acclimation temperature: NAS thermal sensitivity at 21°C acclimation was always higher than that under 15°C acclimation (Figure S2, Tables S9 and S10). For the toads acclimated first at 15°C and then at 21°C, whether they were from low or high altitude, there was no difference in NAS thermal sensitivity between cold and warm acclimation conditions.

Under the acclimation order of first at 21°C then at 15°C, RQ was higher under cold acclimation (1.06 ± 0.04) than under warm acclimation (0.81 ± 0.07) (Figure S3, Tables S11 and S12). However, under the acclimation order of first at 15°C then at 21°C, the RQ was similar under both acclimation conditions (at 21°C–15°C: 1.05 ± 0.08; 15°C–21°C: 1.00 ± 0.08). RQ was negatively associated with the order of the test temperatures.

## 4 Discussion

This study employs an experimental approach to understand how temporal thermal variability modulates ectotherm metabolic plasticity across an altitudinal gradient. We found that RMR acclimation capacity is interactively affected by thermal variability and altitude: metabolic plasticity is higher at low altitudes than at high altitudes, but this variation is strongly suppressed by prior thermal exposure. Unlike RMR, neither altitude nor pre‐thermal variability affected MMR. However, warm acclimation notably enhanced MMR thermal sensitivity. Crucially, thermal variability also significantly altered the type of metabolic substrate utilized. Collectively, these findings on context‐dependent metabolic plasticity and thermal sensitivity provide new insights into broad‐scale physiological responses under climate change.

The altitudinal variation in RMR acclimation capacity in *B. gargarizans* mirrors the latitudinal pattern observed in ectotherms generally (Seebacher et al. 2015), indicating that thermal variability shapes metabolic thermal plasticity across spatial gradients. The loss of RMR plasticity under variable thermal conditions suggests that its associated costs—such as increased ROS production from heightened mitochondrial ATP generation (DeWitt et al. 1998; Norin and Metcalfe 2019)—may outweigh fitness benefits, favoring the evolution of fixed phenotypes (Gunderson and Stillman 2015; Seebacher et al. 2015; Markle and Kozak 2018; Enriquez‐Urzelai et al. 2020; Tan et al. 2024). Notably, compared to the latitudinal gradient (Seebacher et al. 2015), *B. gargarizans* RMR acclimation capacity declines more rapidly with altitude, becoming completely suppressed at 3000 m. Similar suppression has been noted for tropical anurans at similar altitudes (≥ 3000 m) (Navas 1996), implying faster adaptive metabolic divergence along an altitudinal gradient, even with significant gene flow (Keller et al. 2013). Although high‐altitude/high‐latitude populations often exhibit greater plasticity and initial resilience to climate change, ongoing warming may drive larger shifts in both plasticity and selection responses compared to their low‐altitude/low‐latitude counterparts, as seen in *Drosophila subobscura* (Antunes et al. 2024).

It is also worth noting that a recent study found higher RMR plasticity at high versus low latitudes in *Takydromus* lizards, contrasting with the general latitudinal patterns in terrestrial ectotherms (Tsuji 1988; Sun et al. 2022). This suggests that the potential evolution of specific antioxidant damage mechanisms in high‐latitude lizards indicates that the evolution of metabolic plasticity could be closely linked to species' natural history and ecology (Huey et al. 2012; Hu et al. 2019; Zhao et al. 2020).

Consistent with our expectation, both temporal and spatial thermal variability influence metabolic plasticity (Chown and Gaston 2008; Gaston et al. 2009). The detrimental effects of pre‐acclimation thermal variability support the beneficial acclimation hypothesis but contradict the “jack of all trades, master of some” framework. Under global warming, characterized by higher average temperatures and more frequent heat waves, survival depends not only on organism's performance but also on the plasticity of that performance, particularly for low‐altitude/low‐latitude species (Anderson et al. 2022; Jiang et al. 2023). The interactive influence of spatiotemporal thermal variability is further reflected in body condition: at low altitudes, individuals exposed to pre‐acclimation thermal variability exhibited greater mass than those unexposed individuals. This suggests that maintaining metabolic capacity is essential for balancing energetic budgets, providing new insights into ectotherms' macrophysiology.

Unlike RMR, toad MMR is sensitive only to acute temperature changes, not to altitude, acclimation temperature, or pre‐acclimation thermal treatment. This supports the “plastic floors and concrete ceilings” hypothesis (Sandblom et al. 2016). The divergent responses of MMR and RMR also suggest that they may evolve independently (Auer et al. 2017; Norin and Metcalfe 2019). Interestingly, the thermal sensitivity of MMR increased after warm acclimation in both low‐ and high‐altitude populations. If the pattern is generalizable, the enhanced thermal sensitivity of MMR could represent a potential mechanism by which climate warming threatens ectotherm populations' resilience. In the present study, high‐altitude males exhibited lower MMR than their low‐altitude counterparts, contrasting with our previous findings (Zhang et al. 2024). This discrepancy may be potentially due to the different sampling transects belonging to distinct genetic clades with varying sensitivities to thermal variability (Fu 2023; Zhang et al. 2023).

Our results indicate that the toads differ in RQ between cold and warm acclimations only when subjected to stable thermal conditions prior to acclimation. This suggests that temporal thermal variability disrupts both energy expenditure processes and metabolic substrate utilization, potentially altering food choice. The low RQ of 0.81 during cold acclimation suggests a preference for lipid‐based metabolism in cold seasons, such as breeding periods (January–March) or approaching winter dormancy (October), as lipids offer a high energy yield and serve as a long‐term fuel. In contrast, the elevated RQ (1.08) during warm acclimation suggests a shift toward carbohydrate‐based metabolism in warm seasons (active seasons), facilitating rapid energy release to support swift, high‐intensity activities (e.g., escape, striking prey) (Grafe 1996; Donohoe et al. 1998). This thermal environments—dependent substrate utilization aligns with findings on seasonal body composition variations in other anurans (Smith 1976).

A limitation of our experimental design is the absence of replication in the sampling transect, which prevents us from entirely discounting the possibility that these variations in plasticity are solely attributable to population divergence. The pattern observed in the current study aligns with findings from our previous research Tan et al. (2024), thereby supporting our conclusions. The research is confined to two altitude groups and excludes samples from intermediate elevations, limiting our ability to determine whether the observed metabolic differences reflect a progressive gradient response or a threshold effect.

## Author Contributions

**Yuechan Zhang:** conceptualization (equal), investigation (equal), writing – original draft (equal). **Song Tan:** formal analysis (supporting), investigation (supporting). **Jinzhong Fu:** writing – review and editing (supporting). **Jingfeng Chen:** conceptualization (equal), funding acquisition (lead), project administration (lead), resources (lead), writing – original draft (lead), writing – review and editing (lead).

## Conflicts of Interest

The authors declare no conflicts of interest.

## Supporting information


**Figure S1:** The effects of acclimation temperature, acclimation sequence, test temperature, and altitude on body mass of *Bufo gargarizans* . Top left: the high‐altitude group acclimated first at 21°C and then at 15°C. Top right: the high‐altitude group acclimated first at 15°C and then at 21°C. Bottom left: the low‐altitude group acclimated first at 21°C and then at 15°C. Bottom right: the high‐altitude group acclimated first at 15°C and then at 21°C. The blue or red dots represent the predicted value of RMR at 15°C and 21°C acclimations respectively. The blue or red lines are the regression lines based on the predicted body mass.

**Figure S2:** The effects of acclimation temperature, acclimation sequence, test temperature, and altitude on net arobic scope (NAS) of *Bufo gargarizans* . Top left: the high‐altitude group acclimated first at 21°C and then at 15°C. Top right: the high‐altitude group acclimated first at 15°C and then at 21°C. Bottom left: the low‐altitude group acclimated first at 21°C and then at 15°C. Bottom right: the high‐altitude group acclimated first at 15°C and then at 21°C. The blue or red dots represent the predicted value of RMR at 15°C and 21°C acclimations respectively. The blue or red lines are the regression lines based on the predicted NAS.

**Figure S3:** The effects of acclimation temperature, acclimation sequence, test temperature, and altitude on respiratory quotient (RQ) of *Bufo gargarizans* . Top left: the high‐altitude group acclimated first at 21°C and then at 15°C. Top right: the high‐altitude group acclimated first at 15°C and then at 21°C. Bottom left: the low‐altitude group acclimated first at 21°C and then at 15°C. Bottom right: the high‐altitude group acclimated first at 15°C and then at 21°C. The blue or red dots represent the predicted value of RMR at 15°C and 21°C acclimations respectively. The blue or red lines are the regression lines based on the predicted RQ.

**Table S1:** Information of the three sampling sites of *Bufo gargarizans*.
**Table S2:** Comparison of candidate models of body mass in *Bufo gargarizans*.
**Table S3:** The interactive effects of elevation, acclimation temperature, test temperature, and acclimation temperature order on body mass of *Bufo gargarizans*.
**Table S4:** Comparison of candidate models of resting metabolic rate in *Bufo gargarizans*.
**Table S5:** The interactive effects of elevation, acclimation temperature, test temperature, and acclimation temperature order on resting metabolic rate of *Bufo gargarizans*.
**Table S6:** Comparison of candidate models of maximum metabolic rate in *Bufo gargarizans*.
**Table S7:** The interactive effects of elevation, acclimation temperature, test temperature, and acclimation temperature order on the maximum metabolic rate of *Bufo gargarizans*.
**Table S8:** Correlation between standard metabolic rate and maximum metabolic rate in *Bufo gargarizans*.
**Table S9:** Comparison of candidate models of aerobic scope in *Bufo gargarizans*.
**Table S10:** The interactive effects of elevation, acclimation temperature, test temperature, and acclimation temperature order on aerobic scope of *Bufo gargarizans*.
**Table S11:** Comparison of candidate models of respiratory quotient in *Bufo gargarizans*.
**Table S12:** The interactive effects of altitude, acclimation temperature, test temperature, and acclimation temperature order on respiratory quotient of *Bufo gargarizans*.

## Data Availability

Data available from the figshare Repository, https://doi.org/10.6084/m9.figshare.28127840.v1.
