# Supplementary figures and images for: Thermal Variability Modulates Altitudinal Differences in Metabolic Plasticity of the Asiatic Toad
Source: Ecol Evol. 2025 Oct 8;15(10):e72319. doi: 10.1002/ece3.72319 (PMC12508257; doi:10.1002/ece3.72319)

Predicted body mass

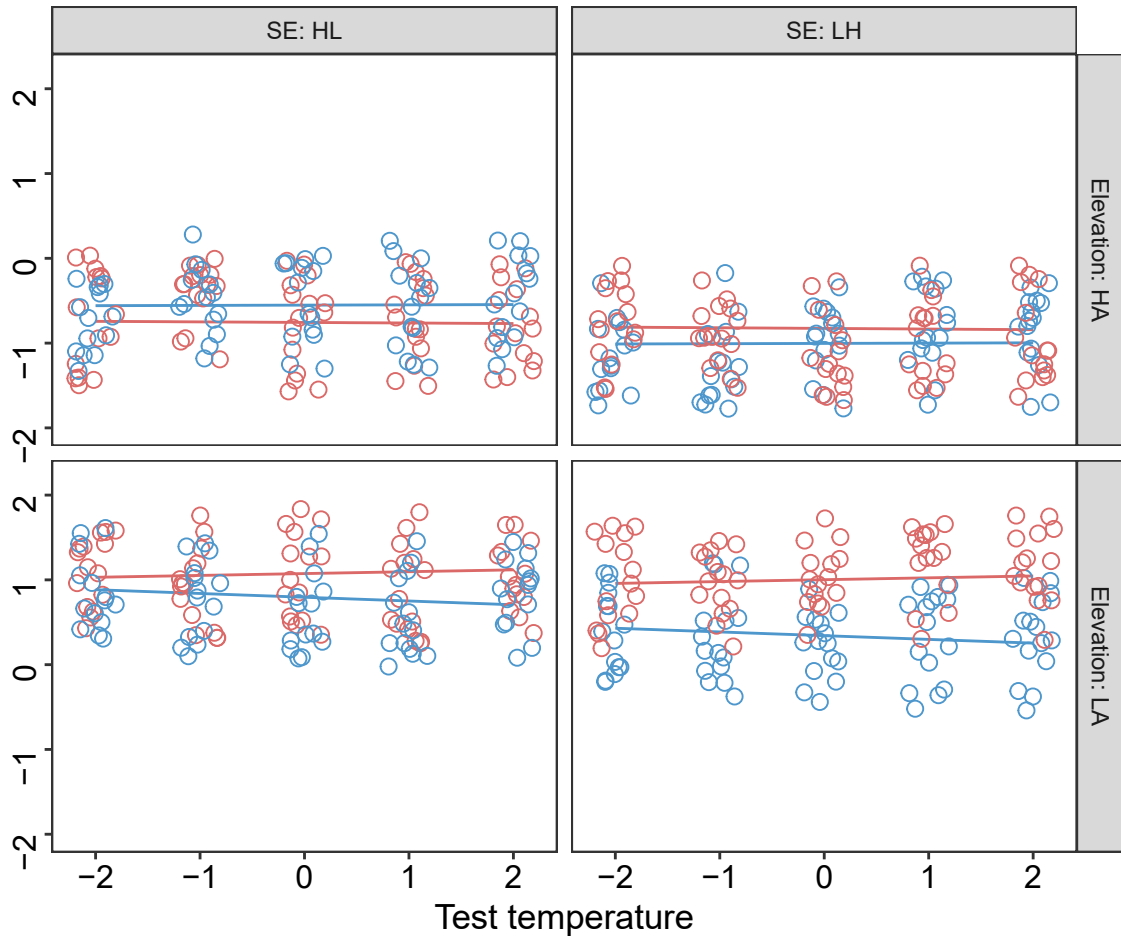

Supplement: Supplementary file 1 — Figure S1: The effects of acclimation temperature, acclimation sequence, test temperature, and altitude on body mass of Bufo gargarizans . Top left: the high‐altitude group acclimated first at 21°C and then at 15°C. Top right: the high‐altitude group acclimated first at 15°C and then at 21°C. Bottom left: the low‐altitude group acclimated first at 21°C and then at 15°C. Bottom right: the high‐altitude group acclimated first at 15°C and then at 21°C. The blue or red dots represent the predicted value of RMR at 15°C and 21°C acclimations respectively. The blue or red lines are the regression lines based on the predicted body mass. [file ECE3-15-e72319-s002.pdf]

Predicted NAS

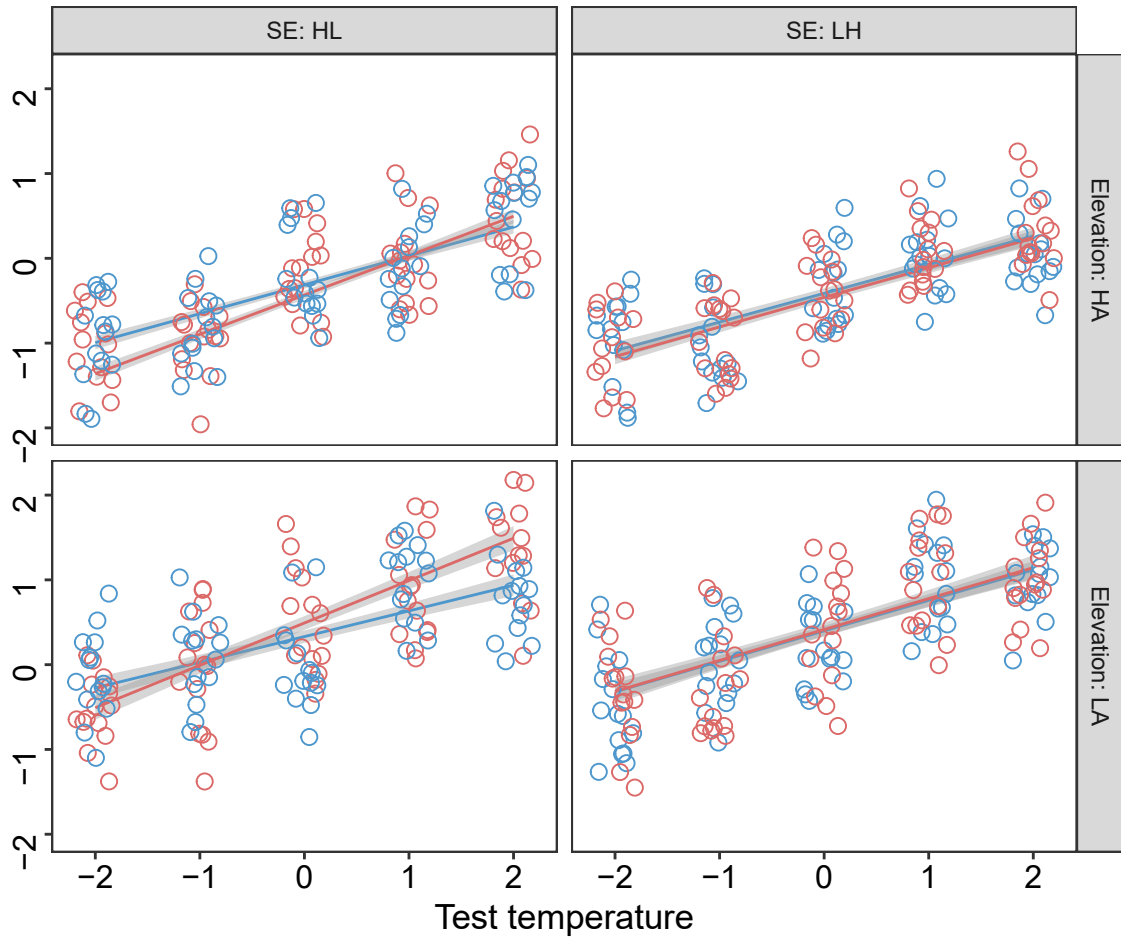

Supplement: Supplementary file 2 — Figure S2: The effects of acclimation temperature, acclimation sequence, test temperature, and altitude on net arobic scope (NAS) of Bufo gargarizans . Top left: the high‐altitude group acclimated first at 21°C and then at 15°C. Top right: the high‐altitude group acclimated first at 15°C and then at 21°C. Bottom left: the low‐altitude group acclimated first at 21°C and then at 15°C. Bottom right: the high‐altitude group acclimated first at 15°C and then at 21°C. The blue or red dots represent the predicted value of RMR at 15°C and 21°C acclimations respectively. The blue or red lines are the regression lines based on the predicted NAS. [file ECE3-15-e72319-s003.pdf]

Predicted RQ

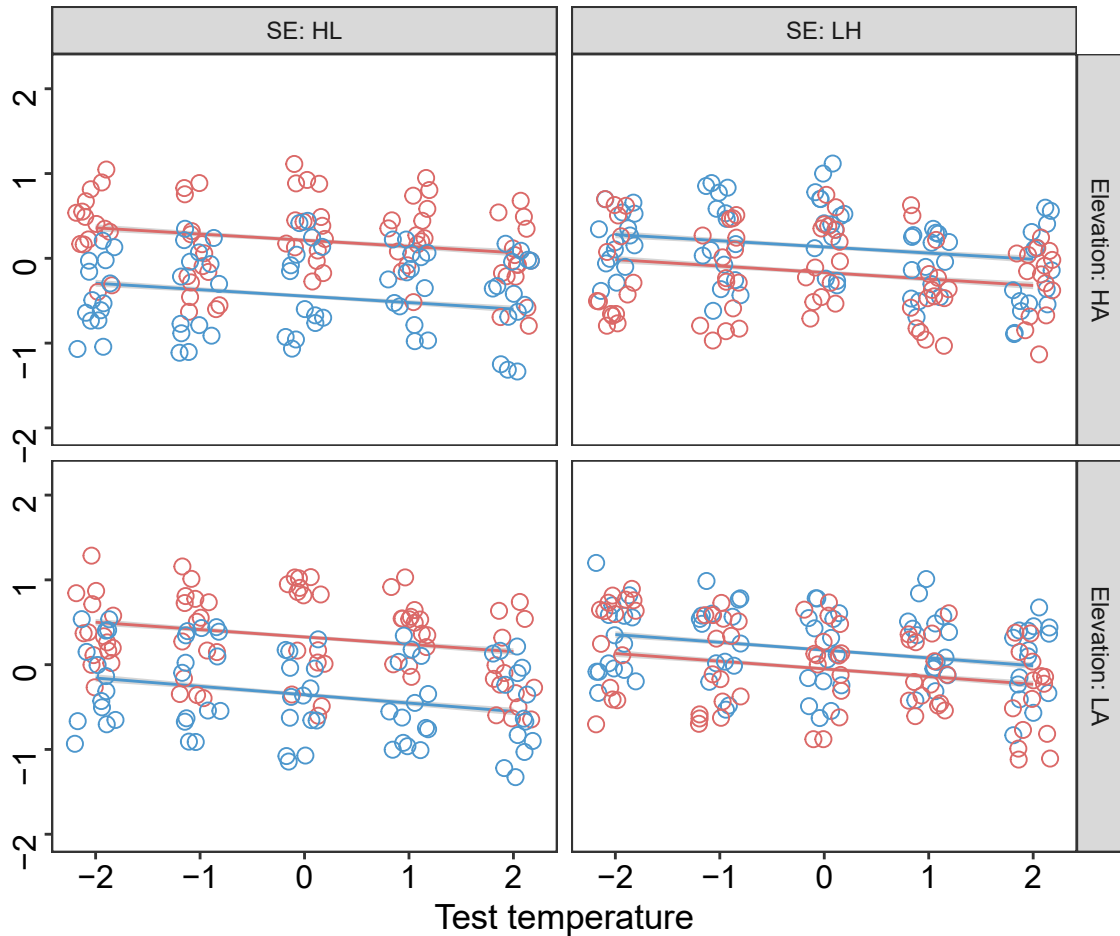

Supplement: Supplementary file 3 — Figure S3: The effects of acclimation temperature, acclimation sequence, test temperature, and altitude on respiratory quotient (RQ) of Bufo gargarizans . Top left: the high‐altitude group acclimated first at 21°C and then at 15°C. Top right: the high‐altitude group acclimated first at 15°C and then at 21°C. Bottom left: the low‐altitude group acclimated first at 21°C and then at 15°C. Bottom right: the high‐altitude group acclimated first at 15°C and then at 21°C. The blue or red dots represent the predicted value of RMR at 15°C and 21°C acclimations respectively. The blue or red lines are the regression lines based on the predicted RQ. [file ECE3-15-e72319-s001.pdf]
