# Supplementary material for: Thermal Variability Modulates Altitudinal Differences in Metabolic Plasticity of the Asiatic Toad
Source: Ecol Evol. 2025 Oct 8;15(10):e72319. doi: 10.1002/ece3.72319 (PMC12508257; doi:10.1002/ece3.72319)
Supplement: Supplementary file 4 — Table S1: Information of the three sampling sites of Bufo gargarizans. Table S2: Comparison of candidate models of body mass in Bufo gargarizans. Table S3: The interactive effects of elevation, acclimation temperature, test temperature, and acclimation temperature order on body mass of Bufo gargarizans. Table S4: Comparison of candidate models of resting metabolic rate in Bufo gargarizans. Table S5: The interactive effects of elevation, acclimation temperature, test temperature, and acclimation temperature order on resting metabolic rate of Bufo gargarizans. Table S6: Comparison of candidate models of maximum metabolic rate in Bufo gargarizans. Table S7: The interactive effects of elevation, acclimation temperature, test temperature, and acclimation temperature order on the maximum metabolic rate of Bufo gargarizans. Table S8: Correlation between standard metabolic rate and maximum metabolic rate in Bufo gargarizans. Table S9: Comparison of candidate models of aerobic scope in Bufo gargarizans. Table S10: The interactive effects of elevation, acclimation temperature, test temperature, and acclimation temperature order on aerobic scope of Bufo gargarizans. Table S11: Comparison of candidate models of respiratory quotient in Bufo gargarizans. Table S12: The interactive effects of altitude, acclimation temperature, test temperature, and acclimation temperature order on respiratory quotient of Bufo gargarizans. [file ECE3-15-e72319-s004.docx]

**Suppl. Table 1** **Information of the three sampling sites of *Bufo gargarizans***

| **Sites** | **coordinates** | **Elevation (m)** |  | **Sample size** | **Mean temperature between**  **May to September (mean±s.e.)*** |
| --- | --- | --- | --- | --- | --- |
| Moxi Ancient town (MA) | 102° 13′ 61.8″ E, 29° 63′ 70.6″ N | 1440m | 2022.1.26 | 30 | 11.9-13.8 ℃ |
| Yumo Road (YR) | 101° 96′ 39.9″ E, 29° 93′ 74.8″ N | 3086m | 2022.4.26 | 27 | 6.8-8.2 ℃ |
| Kangding Cemetery (KC) | 101° 96′ 01.5″ E, 29° 91′ 10.5″ N | 3271m | 2022.4.26 | 3 | 5.4-7.1 ℃ |

* *Climatic variables taken from *WorldClim* database (<http://www.worldclim.org/>). The mean temperature was calculated from the raw data of 2012–2019, which crossed the lifespan of most of the sampled toads.

**Suppl. Table 2 Comparison of candidate models of body mass in *Bufo gargarizans***

| **Candidate models** | **DIC** | **∆DIC** |
| --- | --- | --- |
| Model 1：~ Elev + Ta + Tt + SE + se + Round + Ta*Elev + (1\|ID) | 229.377 | 3.965 |
| Model 2：~ Model 1+ Ta*Tt | 228.087 | 2.675 |
| Model 3：~ Model 1+ Ta*Tt + Elev*Tt | 229.620 | 4.208 |
| Model 4：~ Model 1+ Ta*Tt + Elev*Tt + Elev*Ta*Tt | 225.412 | 0 |

**Notes:** Elev: Elevation; Ta: acclimation temperature; Tt: test temperature; SE: Sequence of acclimation temperature; se: sequence of test temperature; ID: individual identification; * stands for interactive effects. Round: sequence of monitoring body mass during the whole experiment.

**Suppl. Table 3 The interactive effects of elevation, acclimation temperature, test temperature and acclimation temperature order on body mass of *Bufo gargarizans***

| **Variables** | **Posterior mean** | **Lower 95%CI** | **Upper 95%CI** | **pMCMC** |
| --- | --- | --- | --- | --- |
|  | **Fixed effects** |  |  |  |
| Intercept | -0.141 | -0.342 | 0.083 | 0.193 |
| Ta | 0.229 | 0.183 | 0.276 | ＜0.001 |
| SE | -0.256 | -0.553 | 0.034 | 0.076 |
| Tt | 0.020 | -0.041 | -0.001 | 0.045 |
| Se | 0.023 | 0.004 | 0.044 | 0.019 |
| Round | 0.190 | 0.148 | 0.239 | ＜0.001 |
| Elev | -1.578 | -1.867 | -1.302 | ＜0.001 |
| Ta*Elev | -0.483 | -0.576 | -0.395 | ＜0.001 |
| Tt*Elev | 0.013 | -0.019 | 0.046 | 0.416 |
| Ta*Tt | 0.028 | -0.003 | 0.060 | 0.093 |
| Ta*Tt*Elev | -0.075 | 0.139 | -0.017 | 0.012 |
|  | **Random effects** |  |  |  |
| Inter-individual variance | 0.310 | 0.197 | 0.426 |  |
| Intra-individual variance | 0.078 | 0.069 | 0.088 |  |
| Repeatability | 0.812 | 0.730 | 0.856 |  |

**Notes:** * stands for interactive effects.

**Table 4 Comparison of candidate models of resting metabolic rate in *Bufo gargarizans***

| **Candidate models** | **DIC** | **∆DIC** |
| --- | --- | --- |
| Model 1(full model) ：~ Elev + Ta+ Tt+ SE+ Se+ BM + Elev*Tt+ Elev*Ta +Elev*SE +  Ta*Tt + Ta*SE +Tt*SE + Ta*SE*Elev + Ta*SE*Tt +Tt*SE*Ta + Tt*SE*Elev + Elev*Ta*Tt*SE +(1\|ID) | 1241.067 |  |
| Model 2：~ Model 1 - Elev*Ta*Tt*SE | 1238.301 |  |
| Model 3：~ Model 1 - Tt*SE*Elev - Elev*Ta*Tt*SE | 1236.568 |  |
| Model 4：~ Model 1 - Tt*SE*Ta - Tt*SE*Elev - Elev*Ta*Tt*SE | 1236.584 |  |
| Model 5：~ Model 1 - Ta*SE*Tt - Tt*SE*Ta - Tt*SE*Elev - Elev*Ta*Tt*SE | 1235.613 |  |
| Model 6：~ Model 1 - Ta*Tt -Ta*SE*Tt - Tt*SE*Ta - Tt*SE*Elev - Elev*Ta*Tt*SE | 1233.490 |  |
| Model 7：~ Model 1 - Tt*SE - Ta*Tt -Ta*SE*Tt - Tt*SE*Ta - Tt*SE*Elev - Elev*Ta*Tt*SE | 1232.812 |  |

**Notes:** Elev: Elevation; Ta: acclimation temperature; Tt: test temperature; SE: Sequence of acclimation temperature; se: sequence of test temperature; ID: individual identification; * stands for interactive effects.

**Suppl. Table 5 The interactive effects of elevation, acclimation temperature, test temperature, and acclimation temperature order on resting metabolic rate of *Bufo gargarizans***

| **Variables** | **Posterior mean** | **Lower 95%CI** | **Upper 95%CI** | **pMCMC** |
| --- | --- | --- | --- | --- |
|  | **Fixed effects** |  |  |  |
| Intercept | 0.248 | -0.269 | 0.769 | 0.335 |
| Ta | -0.415 | -0.556 | -0.258 | <0.001 |
| SE | -0.389 | -0.598 | -0.153 | <0.001 |
| Tt | 0.363 | 0.315 | 0.411 | <0.001 |
| Se | -0.061 | -0.110 | -0.013 | 0.011 |
| Elev | -1.085 | -1.527 | -0.675 | <0.001 |
| Body mass | -0.011 | -0.164 | 0.153 | 0.909 |
| Ta*Elev | 1.049 | 0.737 | 1.349 | <0.001 |
| Tt*Elev | -0.177 | -0.248 | -0.103 | <0.001 |
| SE*Elev | 0.598 | 0.150 | 1.066 | 0.010 |
| SE*Ta | 0.102 | -0.117 | 0.318 | 0.362 |
| Ta*SE*Elev | -0.793 | -1.233 | -0.353 | <0.001 |
|  | **Random effects** |  |  |  |
| Inter-individual variance | 0.154 | 0.085 | 0.234 |  |
| Intra-individual variance | 0.434 | 0.381 | 0.487 |  |
| Repeatability | 0.263 | 0.157 | 0.355 |  |

**Notes:** * stands for interactive effects.

**Suppl. Table 6 Comparison of candidate models of maximum metabolic rate in *Bufo gargarizans***

| **Candidate models** | **DIC** | **∆DIC** |
| --- | --- | --- |
| Model 1 (full model) ：~ Elev +Ta +Tt+ SE + Se + body mass + Ta*Tt + Ta*SE + Tt*SE + Ta*Elev +Tt*Elev + SE*Elev + Ta*Elev*SE + Ta*SE*Tt + Ta*Tt*Elev + Elev*Tt*SE + Ta*SE*Elev*Tt + (1\|ID) | 923.518 | 6.435 |
| Model 2：~ Model 1 - Ta*SE*Elev*Tt | 921.277 | 4.194 |
| Model 3：~ Model 1 - Ta*Tt*Elev - Ta*SE*Elev*Tt | 921.328 | 4.245 |
| Model 4：~ Model 1 - Ta*Tt*Elev - Elev*Tt*SE - Ta*SE*Elev*Tt | 919.247 | 2.164 |
| Model 5：~ Model 1 - Ta*SE*Tt - Ta*Tt*Elev - Elev*Tt*SE - Ta*SE*Elev*Tt | 920.384 | 3.301 |
| Model 6：~ Model 1 - Ta*Elev*SE - Ta*SE*Tt - Ta*Tt*Elev - Elev*Tt*SE - Ta*SE*Elev*Tt | 920.956 | 3.873 |
| Model 7：~ Model 1- SE*Elev - Ta*Elev*SE - Ta*SE*Tt - Ta*Tt*Elev - Elev*Tt*SE - Ta*SE*Elev*Tt | 920.510 | 3.427 |
| Model 8：~ Model 1 -Tt*Elev - SE*Elev - Ta*Elev*SE - Ta*SE*Tt - Ta*Tt*Elev - Elev*Tt*SE - Ta*SE*Elev*Tt | 919.422 | 2.339 |
| Model 9：~ Model 1- Ta*Elev -Tt*Elev - SE*Elev - Ta*Elev*SE - Ta*SE*Tt - Ta*Tt*Elev - Elev*Tt*SE - Ta*SE*Elev*Tt | 917.083 | 0 |

**Notes:** Elev: elevation; Ta: acclimation temperature; Tt: test temperature; SE: acclimation temperature order; se: test temperature order; BM: body mass; ID: indicates the ID of an individual; * stands for interactive effects.

**Suppl. Table 7 The interactive effects of elevation, acclimation temperature, test temperature and acclimation temperature order on the maximum metabolic rate of *Bufo gargarizans***

| **Variables** | **Posterior mean** | **Lower 95%CI** | **Upper 95%CI** | **pMCMC** |
| --- | --- | --- | --- | --- |
|  | **Fixed effects** |  |  |  |
| Intercept | 0.054 | -0.103 | 0.207 | 0.490 |
| Ta | -0.052 | -0.169 | 0.067 | 0.392 |
| SE | -0.012 | -0.233 | 0.206 | 0.930 |
| Tt | 0.487 | 0.441 | 0.531 | ＜0.001 |
| Se | -0.086 | -0.122 | -0.051 | ＜0.001 |
| Elev | -0.343 | -0.629 | -0.037 | 0.024 |
| Body mass | 0.351 | 0.224 | 0.482 | ＜0.001 |
| SE*Ta | -0.158 | -0.331 | 0.008 | 0.073 |
| SE*Tt | -0.051 | -0.103 | 0.011 | 0.079 |
| Ta*Tt | 0.071 | 0.014 | 0.131 | 0.016 |
|  | **Random effects** |  |  |  |
| Inter-individual variance | 0.165 | 0.099 | 0.247 |  |
| Intra-individual variance | 0.253 | 0.224 | 0.288 |  |
| Repeatability | 0.379 | 0.274 | 0.497 |  |

**Notes:** * stands for interactive effects.

**Suppl. Table 8 Correlation between standard metabolic rate and maximum metabolic rate in *Bufo gargarizans***

| **Variables** | **Posterior mean** | **Lower 95%CI** | **Upper 95%CI** | **pMCMC** |
| --- | --- | --- | --- | --- |
|  | **Fixed effects** |  |  |  |
| RMR | 0.011 | -0.112 | 0.144 | 0.893 |
| MMR | 0.045 | -0.059 | 0.156 | 0.415 |
| BM__RMR_ | 0.194 | 0.063 | 0.337 | 0.009 |
| BM__MMR_ | 0.499 | 0.397 | 0.615 | ＜0.001 |
|  | **Inter-individual random effects** |  |  |  |
| Var__RMR_ | 0.174 | 0.075 | 0.284 |  |
| Var__MMR_ | 0.116 | 0.060 | 0.183 |  |
| Covar__RMR vs MMR_ | 0.028 | -0.029 | 0.093 |  |
|  | **Intra-individual random effects** |  |  |  |
| Var__RMR_ | 0.794 | 0.704 | 0.896 |  |
| Var__MMR_ | 0.654 | 0.578 | 0.736 |  |
| Covar__RMR vs MMR_ | 0.360 | 0.294 | 0.419 |  |

**Notes:** Var represents variance; Covar represents the covariance of intra-individuals or inter-individuals.

**Suppl. Table 9 Comparison of candidate models of aerobic scope in *Bufo gargarizans***

| **Candidate models** | **DIC** | **∆DIC** |
| --- | --- | --- |
| Model 1 (full model) ：~ Elev +Ta +Tt+ SE + Se + body mass + Ta*Tt + Ta*SE + Tt*SE + Ta*Elev +Tt*Elev + SE*Elev + Ta*Elev*SE + Ta*SE*Tt + Ta*Tt*Elev + Elev*Tt*SE + Ta*SE*Elev*Tt + (1\|ID) | 1028.690 | 4.161 |
| Model 2: ~ Model 1- Ta*SE*Elev*Tt | 1026.629 | 2.1 |
| Model 3: ~ Model 1 -Tt*SE*Elev - Ta*SE*Elev*Tt | 1026.590 | 2.061 |
| Model 4: ~ Model 1 - Ta*Tt*Elev - Tt*SE*Elev - Ta*SE*Elev*Tt | 1024.529 | 0 |

**Notes:** Elev: elevation; Ta: acclimation temperature; Tt: test temperature; SE: acclimation temperature order; se: test temperature order; BM: body mass; ID: indicates the ID of an individual; * stands for interactive effects.

**Suppl. Table 10 The interactive effects of elevation, acclimation temperature, test temperature and acclimation temperature order on aerobic scope of *Bufo gargarizans***

| **Variables** | **Posterior mean** | **Lower 95%CI** | **Upper 95%CI** | **pMCMC** |
| --- | --- | --- | --- | --- |
|  | **Fixed effects** |  |  |  |
| Intercept | -0.035 | -0.190 | 0.129 | 0.662 |
| Ta | -0.006 | -0.127 | 0.132 | 0.908 |
| SE | 0.077 | -0.128 | 0.312 | 0.462 |
| Tt | 0.460 | 0.410 | 0.512 | ＜0.001 |
| Se | -0.086 | -0.124 | -0.045 | ＜0.001 |
| Elev | 0.009 | -0.388 | 0.417 | 0.941 |
| BM | 0.460 | 0.326 | 0.604 | ＜0.001 |
| Ta*Elev | -0.181 | -0.445 | 0.095 | 0.165 |
| Tt*Elev | -0.031 | -0.097 | 0.026 | 0.310 |
| SE*Elev | -0.210 | -0.628 | 0.254 | 0.336 |
| SE*Ta | -0.204 | -0.388 | -0.027 | 0.025 |
| SE*Tt | -0.054 | -0.118 | 0.007 | 0.093 |
| Ta*Tt | 0.131 | 0.045 | 0.225 | 0.007 |
| Ta*SE*Elev | 0.493 | 0.110 | 0.843 | 0.015 |
| Ta*SE*Tt | -0.119 | -0.239 | 0.005 | 0.057 |
|  | **Random effects** |  |  |  |
| Inter-individual variance | 0.149 | 0.083 | 0.225 |  |
| Intra-individual variance | 0.302 | 0.267 | 0.342 |  |
| Repeatability | 0.316 | 0.226 | 0.442 |  |

**Notes:** * stands for interactive effects.

**Suppl. Table 11 Comparison of candidate models of respiratory quotient in *Bufo gargarizans***

| **Candidate models** | **DIC** | **∆DIC** |
| --- | --- | --- |
| Model 1 (full model) ：~ Elev +Ta +Tt+ SE + Se + body mass + Ta*Tt + Ta*SE + Tt*SE + Ta*Elev +Tt*Elev + SE*Elev + Ta*Elev*SE + Ta*SE*Tt + Ta*Tt*Elev + Elev*Tt*SE + Ta*SE*Elev*Tt + (1\|ID) | 1611.306 | 5.779 |
| Model 2: ~ Model 1- Ta*SE*Elev*Tt | 1618.165 | 12.638 |
| Model 3: ~ Model 1 -Ta*Tt*SE - Ta*SE*Elev*Tt | 1618.154 | 12.627 |
| Model 4: ~ Model 1 -Tt*SE*Elev -Ta*Tt*SE - Ta*SE*Elev*Tt | 1616.643 | 11.116 |
| Model 5: ~ Model 1 -Ta*Tt*SE - Tt*SE*Elev -Ta*Tt*SE - Ta*SE*Elev*Tt | 1614.670 | 9.143 |
| Model 6: ~ Model 1- Elev*Ta*SE -Ta*Tt*SE - Tt*SE*Elev -Ta*Tt*SE - Ta*SE*Elev*Tt | 1612.829 | 7.302 |
| Model 7：~ Model 1- Tt*SE - Elev*Ta*SE -Ta*Tt*SE - Tt*SE*Elev -Ta*Tt*SE - Ta*SE*Elev*Tt | 1610.845 | 5.318 |
| Model 8：~ Model 1- Elev*SE - Tt*SE - Elev*Ta*SE -Ta*Tt*SE - Tt*SE*Elev -Ta*Tt*SE - Ta*SE*Elev*Tt | 1609.849 | 4.322 |
| Model 8：~ Model 1- Elev*Tt - Elev*SE - Tt*SE - Elev*Ta*SE -Ta*Tt*SE - Tt*SE*Elev -Ta*Tt*SE - Ta*SE*Elev*Tt | 1607.758 | 2.231 |
| Model 9：~ Model 1- Elev*Ta -Elev*Tt - Elev*SE - Tt*SE - Elev*Ta*SE -Ta*Tt*SE - Tt*SE*Elev -Ta*Tt*SE - Ta*SE*Elev*Tt | 1605.845 | 0.318 |
| Model 10：~ Model 1- Ta*Tt - Elev*Ta -Elev*Tt - Elev*SE - Tt*SE - Elev*Ta*SE -Ta*Tt*SE - Tt*SE*Elev -Ta*Tt*SE - Ta*SE*Elev*Tt | 1605.527 | 0 |

**Notes:** Elev: altitude; Ta: acclimation temperature; Tt: test temperature; SE: acclimation temperature order; se: test temperature order; BM: body mass; ID: indicates the ID of an individual * stands for interactive effects.

**Suppl. Table 12 The interactive effects of altitude, acclimation temperature, test temperature, acclimation temperature order on respiratory quotient of *Bufo gargarizans***

| **Variables** | **Posterior mean** | **Lower 95%CI** | **Upper 95%CI** | **pMCMC** |
| --- | --- | --- | --- | --- |
|  | **Fixed effects** |  |  |  |
| Intercept | -0.081 | -0.252 | 0.077 | 0.329 |
| Ta | 0.661 | 0.451 | 0.884 | ＜0.001 |
| SE | 0.116 | -0.134 | 0.341 | 0.322 |
| Tt | -0.034 | -0.095 | 0.032 | 0.298 |
| Se | -0.078 | -0.146 | -0.017 | 0.013 |
| Elev | 0.046 | -0.285 | 0.393 | 0.782 |
| Body mass | 0.087 | -0.079 | 0.248 | 0.288 |
| SE*Ta | -0.958 | -1.255 | -0.652 | ＜0.001 |
|  | **Random effects** |  |  |  |
| Inter-individual variance | 0.115 | 0.063 | 0.183 |  |
| Intra-individual variance | 0.836 | 0.739 | 0.937 |  |
| Repeatability | 0.115 | 0.068 | 0.184 |  |

**Notes:** * stands for interactive effects.
